# First person – Andy Shao

**DOI:** 10.1242/dmm.049708

**Published:** 2022-07-25

## Abstract

First Person is a series of interviews with the first authors of a selection of papers published in Disease Models & Mechanisms, helping early-career researchers promote themselves alongside their papers. Andy Shao is first author on ‘ *Arap1* loss causes retinal pigment epithelium phagocytic dysfunction and subsequent photoreceptor death’, published in DMM. Andy is a resident physician in the lab of Dr Ala Moshiri MD, PhD at School of Medicine, UC Davis, Sacramento, CA, USA, investigating the molecular mechanisms of heritable retinal disorders.


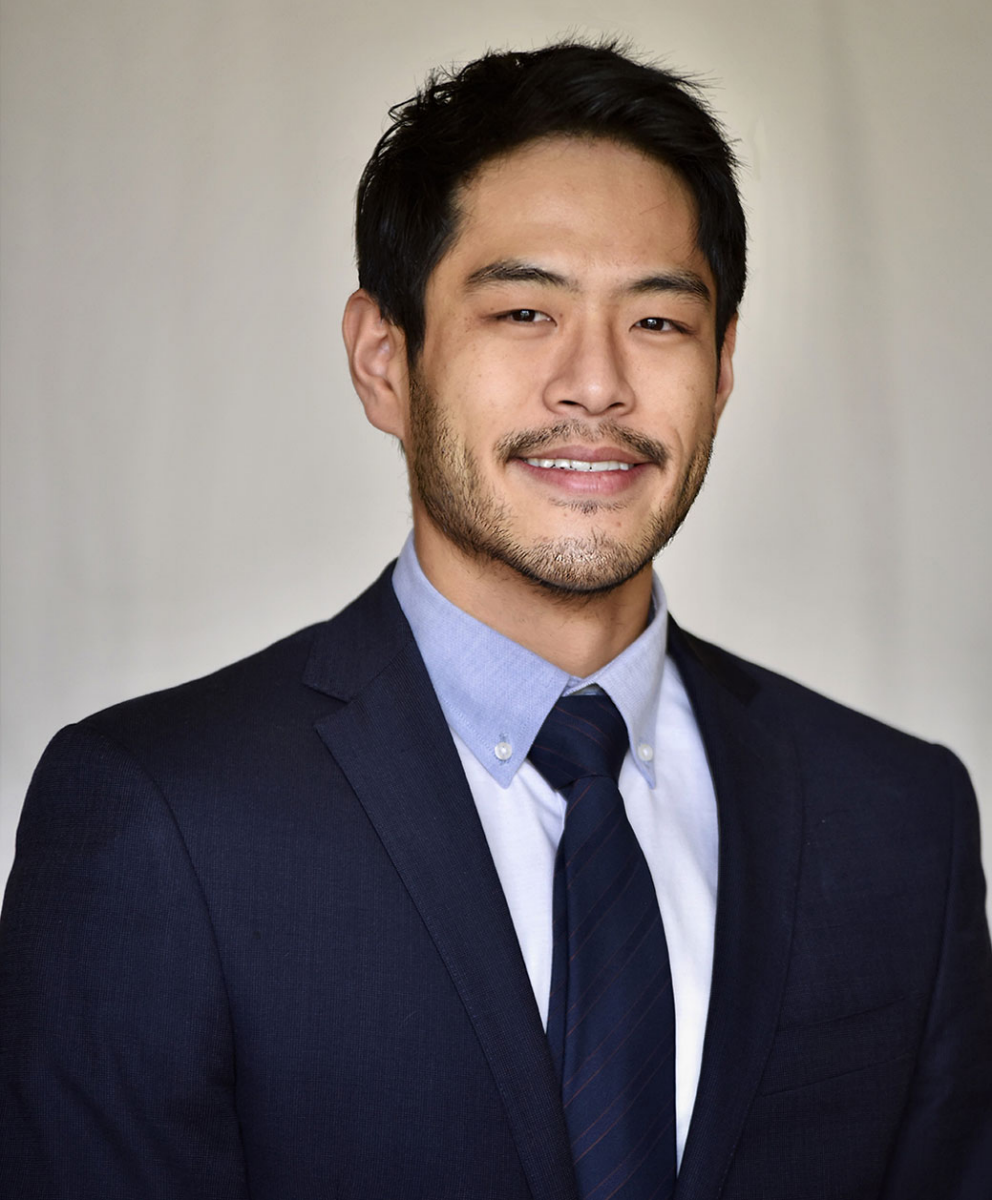


**Andy Shao**

**How would you explain the main findings of your paper to non-scientific family and friends?**

Retinitis pigmentosa (RP) is a genetic disease that causes irreversible blindness due to its effects on the retina, the light-sensing portion of our eyes. Our lab has previously created a mouse missing a key gene known as *Arap1*. Curiously, these mice had abnormalities in their retinas very similar to humans with RP. However, *Arap1* is a gene for which its role in maintaining eye health had not been well explored. In this paper, we sought to find an explanation as to why loss of *Arap1* caused changes in mouse retinas similar to those seen in human RP patients. Our research has revealed that *Arap1* plays an essential role in ensuring the proper function of the retinal pigment epithelium (RPE). The RPE is a layer of cells in our eyes that governs many important jobs that maintain a healthy environment for the various cells in our retinas to grow and function. Loss of *Arap1* caused the RPE to lose much of its ability to consume and clear debris buildup from the retina, causing the death of retinal cells and subsequent vision loss.

“Our research has revealed that *Arap1* plays an essential role in ensuring the proper function of the retinal pigment epithelium.”

**What are the potential implications of these results for your field of research?**

Though it is well known that RPE consumes retinal debris through a process called phagocytosis, knowledge of which proteins help to regulate this complex process is incomplete. Our research has revealed that the gene *Arap1*, which had not been well studied in the context of the eye, is an essential component of this process. Given that Arap1 governs a broad range of functions, our next steps would be to deduce which of these functions are actively participating in RPE phagocytosis. From there, we can better deduce which Arap1-interacting proteins are participating in the phagocytosis pathway and potentially broaden our genetic screens for RP-causative genes.

**What are the main advantages and drawbacks of the model system you have used as it relates to the disease you are investigating?**

The house mouse (*Mus musculus*) is integral to *in vivo* gene and protein research, as was seen in our research. Logistically speaking, working with mice is extremely convenient. Their breeding and turnover rates are rapid, promoting ease of colony generation and planning of age-sensitive experiments. Given that ensuring tissue section quality can often be a finnicky process, the large litters we were able to process for preservation ensured that we had ample tissue for experimental analysis. Additionally, the high degree of human–mouse genome similarity has always been a relative strength of mouse models, though the variance still remains a concern. Of course, there exist notable physiologic differences between mouse and human eyes. Importantly, the photoreceptor composition of mouse retinas is almost exclusively rods, given that they are nocturnal animals.

**Figure:**
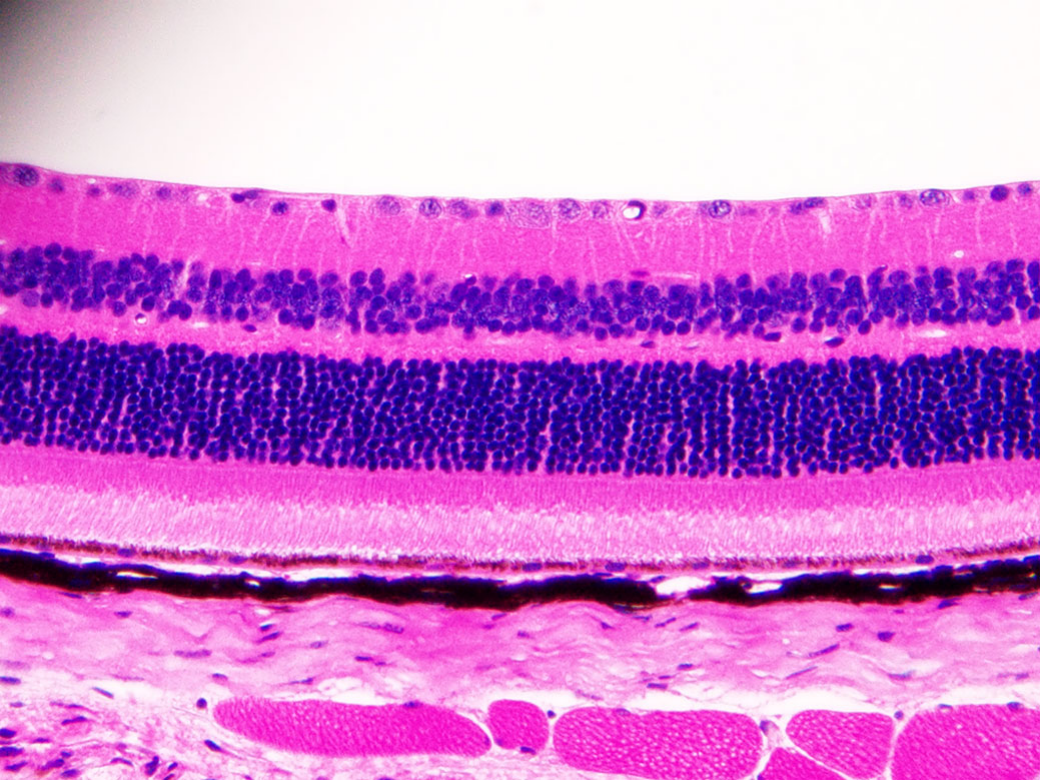
Haematoxylin and Eosin-stained retinal section from a *Glast-Cre* mutant mouse.

**What has surprised you the most while conducting your research?**

This project often reminded me the adage “you don't know what you don't know” again and again. However, I was astounded at how invaluable a resource that forums such as ResearchGate became for me. I would often run into roadblocks during my experiments and have no idea of where to begin the troubleshooting. However, online discussions of similar issues regularly gave me a strong sense of where to take my initial experiments. It really is an incredible thing to see how far information access has come over the last several decades and how it's come to affect academic research.

**Describe what you think is the most significant challenge impacting your research at this time and how will this be addressed over the next 10 years?**

Knowledge of the RPE phagocytosis pathway is incomplete, making it difficult to determine the potential roles of Arap1 and its interactants in this complex process. However with further research, newly elucidated molecular mechanisms can help to bridge these gaps in knowledge to clarify the functions of candidate pathway participants and reveal new candidate interactants.

**What changes do you think could improve the professional lives of early-career scientists?**

Personally, financial limitations were some of the more significant barriers I had to face. My home institution does not have an ophthalmology department, which made doing research in my field of interest difficult. I was fortunate to have UC Davis nearby, but even with the proximity it was still difficult to allocate funding for a research year. Especially knowing that my medical school loans would begin accruing interest on my gap year, limited options for deferment and the lack of federal loan options to fund my research year made finances a significant concern. Though expansion of federally sourced grants for short-term academic endeavours would be great, I believe that easing access to loan deferment and federal loan options for students wishing to pursue academic gap years are small changes that would improve academic accessibility to students from smaller institutions.

“It really is an incredible thing to see how far information access has come over the last several decades and how it's come to affect academic research.”

**What's next for you?**

I am currently entering the intern year of my ophthalmology residency at UC Davis. Though these next 4 years I'll be largely focused on developing and honing my surgical and clinical skills, I hope to continue research with whatever time I can find.
